# The British Nurses' Association and the Medical Council

**Published:** 1889-12-21

**Authors:** Bedford Fenwick, Catherine J. Wood

**Affiliations:** Hon. Secretary; Secretary


### The British Nurses' Association and the Medical Council.

The following is the memorial submitted to the General Council by the British Nurses' Association, which was dated November 20, 1889:

### A GENERAL REGISTRATION OF MIDIVES AND NURSES.

1. The value of skilled attendance upon women during and after labour, and in the treatment of accident or disease, is generally admitted.

2. It would, therefore, seem to be of prime importance that persons who undertake the duties of midwife or nurse should be qualified to discharge their respective duties efficiently, and, moreover, it would certainly be well that they should be under some professional control.

3. It is a well-known fact, however, that at present, any woman, although she may be destitute of knowledge or of moral character, or of both, can without let or hindrance term herself a trained midwife or nurse; can obtain employment in either capacity, and bring about much danger to the sick and discredit to the calling. Nor is there any means of preventing any certificated midwife or nurse who, by drunkenness, theft, or even graver offences, has proved herself unworthy of trust from again and again bringing disgrace to her fellow-workers, and upon the institution whose certificate she is nevertheless able to produce.

4. To meet these undoubted objections the British Nurses' Association suggests that the system of registration of trained midwives and nurses should be carried out, and to that end has proposed the following scheme: (*a*) That a Registration Council should be formed, to which every training school for midwives and nurses in the United Kingdom might be invited to send one member of its medical or nursing staff to represent its views and wishes, upon which the association, as comprising one-fifth of the number of nurses now estimated to be at work, might be represented by the members of its executive committee, and to which other members might be specially added. That this body should meet once or twice a year and decide upon all necessary rules and regulations, and be, in short, the governing body of the scheme. (*b*) That the council should, from its members, appoint a Registration Board of about twenty-four persons to meet as often as requisite to supervise the details of the work, and be, in short, the executive body of the scheme. (*c*) That separate and distinct registers for midwives and for nurses should be forthwith opened and continued. (*d*) That for a limited "period of grace" any person who could produce proof satisfactory to the board that she had been engaged for three years for payment in attendance might be registered, but that thereafter only those who hold a certificate from an obstetrical examining body or a recognised hospital, or could produce other proof satisfactory to the Registration Board of their technical knowledge and efficiency and of their moral character, should be enrolled upon the register. Furthermore, that the board should, subject to a right of appeal to the Registration Council, have the absolute power to remove temporarily, or permanently, from the registers the name, with the qualification, of any midwife or nurse who may show herself to be unworthy of trust.

5. The cardinal principle for which the British Nurses' Association has striven, in the face of enormous opposition, is that the control of midwives and nurses should remain in the hands of the medical and nursing professions, and that this question of registration should be dealt with solely by professional authorities themselves. The association, therefore, submits this scheme to the General Medical Council, and respectfully seeks the advice, support, and assistance of that influential body in the matter. It would venture especially to ask whether it would be advisable that a certain number of representative medical men, obstetric physicians, and midwives should be requested to act as members of the Registration Council, and *ex officio* of the board. And, if so, whether, in the opinion of the General Medical Council, it would be better that these members should be elected by the Registration Council, or that they should be appointed by some outside authority. Finally, if the latter course be considered the more advisable, would the General Medical Council be willing to undertake the duty of nominating such representatives, as such action upon its part would be gratefully accepted.

(Signed) Bedford Fenwick, M.D., Hon. Secretary.
Catherine J. Wood, Secretary.

